# Chicago Dental Society—Resolutions on the Death of Drs. William H. Atkinson and James W. White

**Published:** 1891-07

**Authors:** 


### CHICAGO DENTAL SOCIETY—RESOLUTIONS ON THE DEATH OF DRS. WILLIAM H. ATKINSON AND JAMES W. WHITE.

At the meeting of the Chicago Dental Society, Tuesday evening, May 5, 1891, the following resolutions on the death of Dr. William H. Atkinson, of New York, were adopted:

WHEREAS, The Chicago Dental Society having learned of the death of Dr. William H. Atkinson, of New York, one of the most eminent, learned, and best known members of the dental profession, therefore, be it

*Resolved*, That in the death of Dr. Atkinson the members of this Society feel a sense of personal bereavement in the loss of a much loved and conspicuously useful member of the profession, and while we bow in humble submission to the Divine will, we desire to express our sorrow in his final exit to the unknown land beyond this world of ours.

*Be it further resolved*, That the Secretary transmit to the bereaved family of Dr. Atkinson a copy of these resolutions, and that a copy be furnished the DENTAL JOURNAL for publication.

J. H. CROUSE,

A. W. HARLAN,

W. W. ALLPORT,

*Committee.*

THE following resolution, by Dr. Allport, in regard to the death of Dr. White, was passed by the Chicago Dental Society:

WHEREAS, It hath pleased the Creator and final disposer of all things to remove from this world Dr. James W. White, of Philadelphia; and

WHEREAS, It is fitting that this Society should make some record of its appreciation of his virtues and of his useful life, therefore,

*Resolved*, That in the death of Dr. White dental journalism has lost its ablest editor, the business world a member of sterling integrity, the unfortunate and needy a practical philanthropist, and the church an exemplar of the nobility of a liberal Christian religion.

*Resolved*, That in their affliction we extend to his bereaved family our sincere sympathy, and with reverent humility we commend them to Him who has promised to be "the friend of the widow and the fatherless" and "a real present help in the time of trouble."

*Resolved*, That a copy of these resolutions be transmitted to the family of the deceased, and sent to the *Dental Cosmos* and other dental journals for publication.

